# Preparation of Multicolour Solid Fluorescent Carbon Dots for Light-Emitting Diodes Using Phenylethylamine as a Co-Carbonization Agent

**DOI:** 10.3390/ijms231911071

**Published:** 2022-09-21

**Authors:** Yulong An, Can Liu, Yan Li, Menglin Chen, Yunwu Zheng, Hao Tian, Rui Shi, Xiahong He, Xu Lin

**Affiliations:** 1 National Joint Engineering Research Center for Highly-Efficient Utilization Technology of Forestry Resources, Southwest Forestry University, Kunming 650224, China; 2 Key Laboratory for Forest Resources Conservation and Utilization in the Southwest Mountains of China, Ministry of Education, Southwest Forestry University, Kunming 650224, China; 3 Agro-Products Processing Research Institute, Yunnan Academy of Agricultural Sciences, Kunming 650000, China

**Keywords:** carbon dots, solid fluorescence, solvent control, multicolour, optoelectronic applications

## Abstract

Carbon dots (CDs), as a new type of photoluminescent nanomaterial, have attracted extensive attention in various fields because of their unique luminescence properties. However, CDs will exhibit fluorescence quenching in the solid state or aggregate state, which limits their application. In this paper, a unique strategy is proposed to regulate solutions to achieve multicolour fluorescence of CDs in the solid state. We report the successful preparation of orange, green and blue solid fluorescent CDs using citric acid, urea and phenylethylamine as precursors and methanol, ethanol and water as solvents, respectively. The solid-state fluorescence of CDs may be caused by the linkage of the phenylethyl structure to the surface of CDs during formation, which effectively disperses the CDs and prevents π–π interactions between graphitized nuclei. Meanwhile, multicolour solid fluorescent CDs are realized by adjusting the solvent in the preparation process. Based on the excellent fluorescence properties of CDs, orange, green and blue light-emitting diodes (LEDs) are prepared. A white LED (WLED) can be obtained by mixing the three colours of solid fluorescent CDs, which shows the application potential of CDs in display lighting equipment.

## 1. Introduction

Carbon dots (CDs), as promising photoluminescent nanomaterials, have wide application prospects in biological imaging, sensors and lighting equipment due to their unique luminescence properties [1,2,3,4,5]. Compared to conventional semiconductor quantum dots, CDs have the advantages of a simple preparation process, abundant raw materials, low toxicity, good biocompatibility and easy surface functionalization [6,7,8]. Since Xu found carbon nanoparticles with fluorescence for the first time in the separation and purification of single-walled carbon nanotubes by electrophoresis in 2004 [9], the study of fluorescent CDs has attracted increasing attention. Since there are many raw materials and a variety of preparation methods available in the CD preparation process, various CDs have been prepared [10,11,12,13,14].

Nevertheless, there are still many limitations for synthetic CDs that have good fluorescence properties and even multicolour luminescence. Most CDs can only show fluorescence in the dispersed state, which is their main defect. In the solid state, due to excessive Förster resonance energy transfer and π–π stacking interactions, aggregation-induced fluorescence quenching will occur, which greatly inhibits the application of CDs in various fields [15,16,17,18]. To solve the above shortcoming of CD fluorescence quenching, some researchers have performed much research to study the solid fluorescence of CDs. Specifically, Lei et al. [19] prepared blue and red solid fluorescent CDs by using citric acid as a carbon source and formamide as a nitrogen source. Wei et al. [20] prepared CDs using citric acid, urea and sodium hydroxide as precursors and achieved solid fluorescence of CDs with a quantum yield (QY) of 75.9% by embedding CDs in a NaOH crystal matrix in situ to block the self-quenching of CDs. Shen et al. [21] embedded CDs in situ in a trisodium citrate crystal matrix, and the CDs presented tuneable solid fluorescence emission from green to yellow, with a high QY up to 21.6%. Liu’s team [22] prepared green solid fluorescent CDs with citric acid and branched polyethylenimine and successfully fabricated white light-emitting diodes (WLEDs). However, the preparation of solid fluorescent CDs always has the disadvantages of poor stability, single fluorescence emission and complex preparation methods. Meanwhile, the reported solid fluorescent CDs usually achieve solid fluorescence through embedding in a crystal matrix, so a simple and effective method to prepare more stable CDs with multicolour solid fluorescence emission must be developed [23,24,25,26].

Herein, we propose a solvent-regulated strategy to adjust the fluorescence colour of CDs in the solid state and realize the preparation of solid fluorescent CDs in the full spectrum. Multicolour solid fluorescent CDs were prepared by a solvothermal method with citric acid, urea and phenylethylamine as precursors and methanol, ethanol and water as solvents. The optical properties and morphologies of the prepared solid fluorescent CDs were analysed by modern analytical characterization techniques. Combined with the above characterization analysis, the solid fluorescence mechanism of CDs was studied, and the solvent regulation was confirmed to realize multicolour solid fluorescence of CDs. Orange, green and blue LEDs were prepared by coating CDs on ultraviolet LED chips with a wavelength of 365 nm. WLEDs could be obtained by mixing the three colours of solid fluorescent CDs, which showed the great application potential of CDs in display lighting equipment. This study opens a new way for simple, green and low-cost preparation of polychromatic solid fluorescent CDs and further proves that CDs have great application potential in the field of optoelectronic devices.

## 2. Results

**Figure 1:**
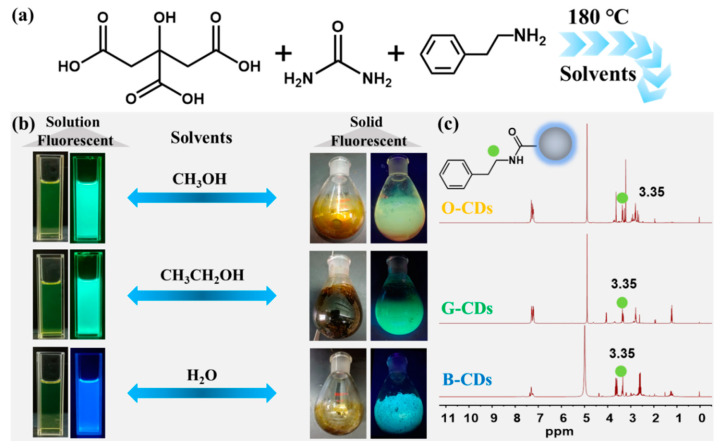
(**a**) Schematic illustration of the preparation of solid-state fluorescent CDs. (**b**) Actual photos of fluorescent CDs with different colour emissions. (**c**) ^1^H NMR spectra of CDs in CD_3_OD.

Solid fluorescent CDs were prepared by a one-step solvothermal method using citric acid as a carbon source, urea as a nitrogen source and phenylethylamine as a co-carbonization agent. We consider that the phenylethyl structure is brought to the CD surface through the reaction between the carboxyl group on citric acid and the amino group on phenylethylamine to physically separate the CDs, inhibit the fluorescence quenching caused by the aggregation of CDs, and realize solid fluorescence of CDs. Firstly, in order to verify this idea, we selected reaction systems with and without phenylethylamine, respectively, and prepared two groups of CDs in water, in which the CDs prepared without phenylethylamine could not display solid fluorescence (Figure S1). Therefore, we chose phenylethylamine as the reaction system of the co-carbonation agent. Through a large number of solvent regulation experiments, methanol, ethanol and water as reaction solvents were found to enable the preparation of multicolour solid fluorescent CDs. The resulting CDs show bright orange (O-CDs), green (G-CDs) and blue (B-CDs) fluorescence in the solid state under UV light (Figure 1a,b and Table S1). The CDs were characterized by ^1^H-NMR, as shown in Figure 1c. The NMR data show that the signal of the H (green mark) on the α-carbon is observed in the three CDs. In addition, the three CDs show the NMR signal of the H on the benzene structure between 7 and 8, which also means that through the chemical reaction between the carboxyl group of citric acid and the amino group of phenylethylamine, the phenylethyl group is successfully linked to the surface of the CDs.

**Figure 2:**
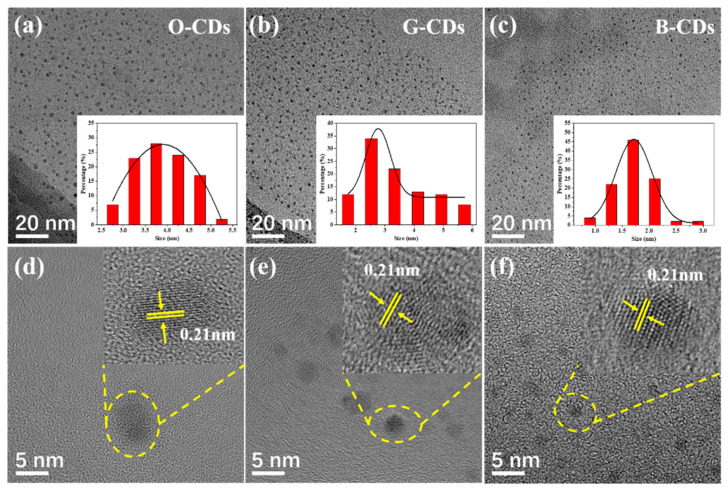
(**a**–**c**) TEM images of O-CDs, G-CDs and B-CDs. Insets: particle size distribution. (**d**–**f**) HRTEM images of O-CDs, G-CDs and B-CDs.

The O-CDs, G-CDs and B-CDs were characterized by transmission electron microscopy (TEM), as shown in Figure 2. We can clearly see that the CDs exhibit a uniform spherical particle shape and good monodispersity. The average particle sizes of O-CDs, G-CDs and B-CDs are 3.75 ± 0.40 nm, 2.72 ± 0.51 nm and 1.75 ± 0.14 nm, respectively, by measuring the particle sizes of 120 CD particles in TEM images, and the particle size of the CDs is positively correlated with the fluorescence. With increasing particle size of the CDs, the CD fluorescence redshifts, and the quantum size effect may be one of the factors leading to the fluorescence difference of the CDs. Figure 2d–f shows high-resolution TEM (HRTEM) images of O-CDs, G-CDs and B-CDs, further showing 2D lattice fringes of the CDs with a lattice spacing of 0.21 nm, close to the (100) diffraction plane of graphite carbon, indicating that the synthesized CDs crystallized well [27,28,29,30].

**Figure 3:**
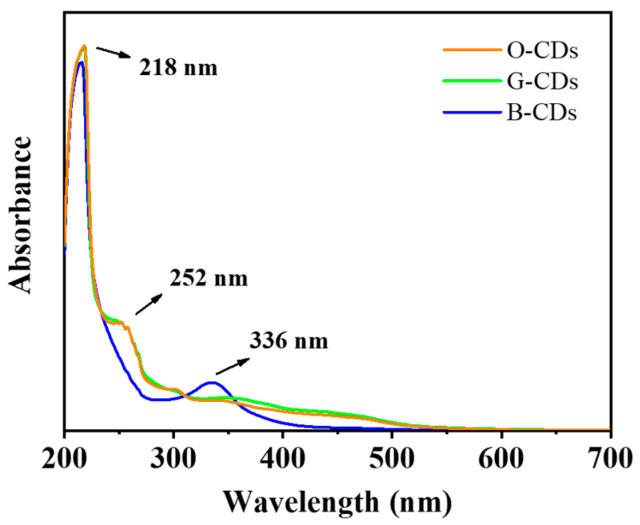
UV/vis absorption spectra of O-CDs, G-CDs, and B-CDs in ethanol solution (*c* = 0.1 mg/mL).

UV—visible absorption spectra of O-CDs, G-CDs and B-CDs in ethanol solution were obtained. As shown in Figure 3, in addition to common ultraviolet absorption near 210 nm, the three CDs have different independent absorption peaks. The absorption peaks of O-CDs and G-CDs at approximately 250 nm are the significant absorptions due to the intrinsic state (π–π*) transition of the aromatic sp^2^ domain (C=C). In addition, the absorption band of B-CDs at 345 nm is caused by the n–π* transition of C=O/C=N-related bonds [31,32,33,34]. Meanwhile, the absorption of O-CDs and G-CDs at 400–500 nm is caused by the surface functional groups of the CDs.

**Figure 4:**
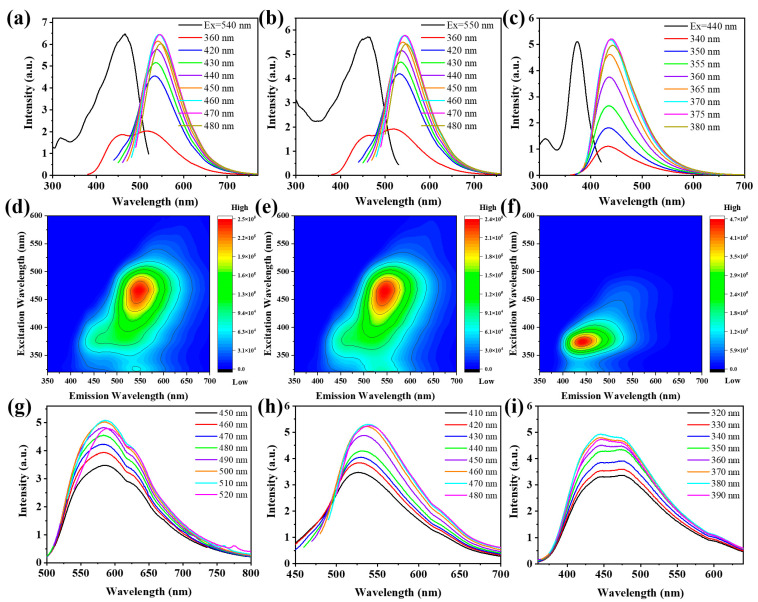
(**a**–**c**) Excitation, emission and (**d**–**f**) 3D spectra of O-CDs, G-CDs and B-CDs in ethanol solution (*c* = 0.1 mg/mL). (**g**–**i**) PL spectra of O-CDs, G-CDs and B-CDs in the solid state.

Photoluminescence (PL) spectra and three-dimensional (3D) fluorescence spectra of O-CDs, G-CDs and B-CDs in ethanol solution are shown in Figure 4a–f. The three CDs show different PL characteristics. The fluorescence of O-CDs and G-CDs is green with fluorescence centres at 549 nm and 543 nm, respectively. The PL of B-CDs is blue with a fluorescence centre at 440 nm. The fluorescence emission spectra show that the fluorescence of the CDs has no obvious excitation wavelength dependence, and the excitation spectra show that the optimal excitation wavelengths of the CDs are 460, 460 and 370 nm. The emission-independence of carbon dots is attributed to the fact that both the size and the surface state of the sp^2^ clusters contained in its structure are uniform [35]. The 3D spectra are consistent with the PL spectra, and the 3D spectra show that there is a single emission centre for all three CDs (Figure 4d–f). In addition, the PLQYs of O-CDs, G-CDs and B-CDs in solution are 36.7, 69.6 and 67.3%, respectively (Table 1). In the solid state, O-CDs, G-CDs and B-CDs show orange, green and blue fluorescence with fluorescence centres of 585, 538, and 445 nm at the optimal excitation wavelengths of 510, 470, and 380 nm, respectively (Figure 4g–i). The fluorescence QYs of O-CDs, G-CDs and B-CDs are 13.9%, 35.7% and 2.6%, respectively. Compared with the fluorescence in solution, the QY of the three CDs significantly decreases. Compared with those reported in the literature, the CDs prepared by our one-step method have higher solid fluorescence QY and show a simpler preparation process for multicolour solid fluorescent CDs (Table S2).

**Figure 5:**
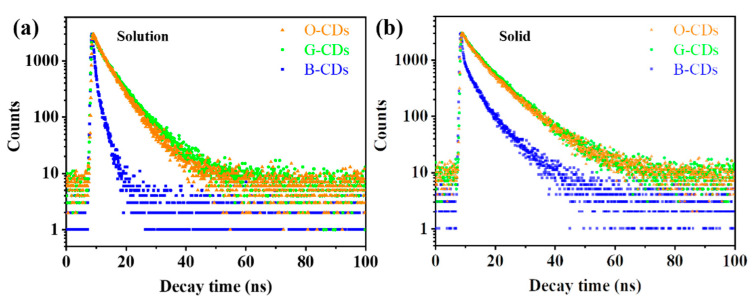
Fluorescence lifetime characterization of O-CDs, G-CDs and B-CDs in ethanol solution (*c* = 0.1 mg/mL) (**a**) and in the solid state (**b**).

In addition, the fluorescence lifetimes of the three CDs were characterized in solution and in the solid state (Figure 5). The fluorescence lifetimes of O-CDs, G-CDs and B-CDs are 7.73, 7.87 and 4.57 ns in solution and 5.03, 5.74 and 1.28 ns in the solid state, respectively. The fluorescence quantum efficiency and lifetime of the solid B-CDs are significantly reduced compared to those of the solution B-CDs, which means that the quenching behaviour caused by the π–π interaction is stronger than that for the other two CDs.

**Figure 6:**
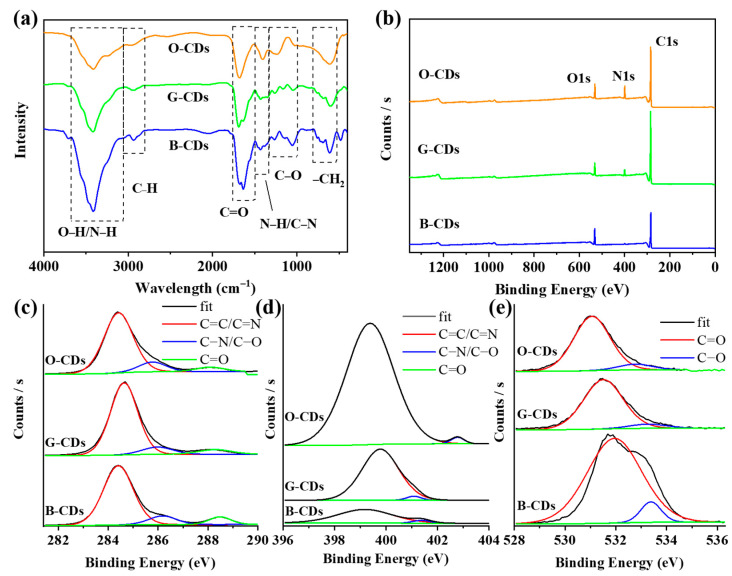
(**a**) FTIR spectra of CDs. (**b**) XPS survey spectra and (**c**–**e**) high-resolution C1s and O1s spectra of CDs.

The surface composition of CDs was characterized by Fourier transform infrared (FTIR) spectroscopy. According to the FTIR results (Figure 6a), the strong characteristic peaks of O-CDs, G-CDs and B-CDs at approximately 3417 cm^−1^ show that there are abundant O-H/N-H groups, and the absorption peaks at 2937 cm^−1^ may be caused by C-H bond vibrations. This indicates that through the reaction between the carboxyl group of citric acid and the amino group of phenylethylamine, the alkyl structure is successfully connected to the surface of CDs, thus blocking the self-quenching of the solid-state fluorescence of CDs [36]. In addition, other vibrational bands corresponding to functional groups such as C=O, N–H, C–N, C–O and –CH_2_ and other chemical bonds are also observed, corresponding to five characteristic absorption peaks at 1680, 1405, 1268, 1046 and 602 cm^−1^, respectively [37,38].

To further explore the composition of the CDs, we performed X-ray photoelectron spectroscopy (XPS) (Figure 6b). The XPS spectra show that the CDs contain three elements, C (284.8 eV), N (398.8 eV) and O (531.8 eV) [39,40,41]. Table 2 shows that the main element of these CDs is carbon, with a small amount of nitrogen and oxygen doping. The nitrogen contents of O-CDs, G-CDs and B-CDs show a decreasing trend, especially the N content of B-CDs, which is the lowest. The high-resolution XPS spectra of O-CDs, G-CDs and B-CDs were analysed (Figure 6c–e). The analytical C1s peaks at 284.7, 286.2 and 288.6 eV indicate the presence of C=C/C=N, C-N/C-O and C=O, respectively [42]. In the O1s spectra of the CDs, the two peaks at 532.0 eV and 533.5 eV belong to C=O and C–O, respectively [43]. The C=C bond contents of O-CDs and G-CDs are higher than that of B-CDs, indicating that the conjugated structures are larger than that of B-CDs. Therefore, O-CDs and G-CDs show green fluorescence in solution, while B-CDs show blue fluorescence.

**Figure 7:**
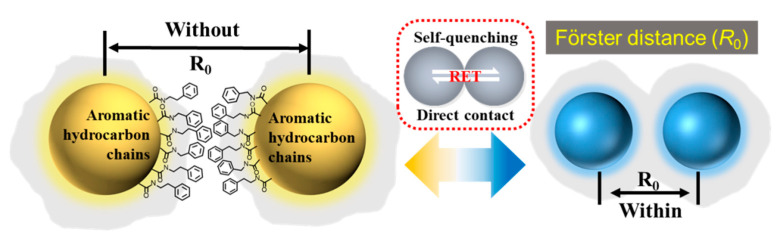
Schematic diagram of the solid-state fluorescence mechanism of CDs.

Similar to the fluorescence quenching phenomenon caused by aggregation in a high-concentration CD solution, there is no dispersion medium in solid CDs, which makes the light-emitting centres of CDs directly contact, leading to the π–π interaction or excessive Förster resonance energy transfer, thus resulting in self-quenching of the fluorescence of solid CDs [44,45,46]. Therefore, introducing functional groups with steric hindrance effects into CDs to achieve the physical separation of CDs is a good strategy. Through the reaction between the carboxyl group of citric acid (CA) and the amino group of phenylethylamine, phenylethylamine is connected to the surface of CDs, forming a “protective shell”. The existence of the “protective shell” plays the role of physically separating adjacent CDs and realizes solid-state fluorescence (Figure 7). CDs prepared by solvent conditioning show different fluorescence colours in the solid state. Combined with TEM and XPS analysis, the particle sizes and nitrogen contents of CDs prepared in methanol, ethanol and water show a downward trend. The quantum size effect may play a role in the fluorescence regulation of CDs. In addition, the nitrogen content also has a certain impact on the fluorescence of CDs. An increase in nitrogen makes the surface state of CDs uniform, reduces their energy level and band gap, and causes a redshift of the emission wavelength of CDs [47].

**Figure 8:**
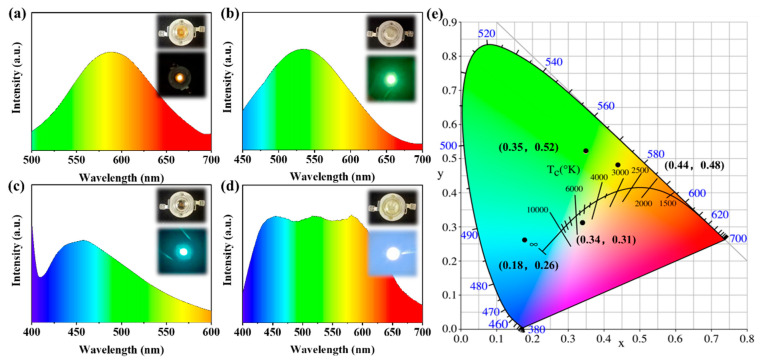
(**a**–**d**) EL emission spectra of the (**a**) O-LED, (**b**) G-LED, (**c**) B-LED and (**d**) WLED. The insets show optical images of the LEDs in the “off” (**top**) and “on” (**bottom**) states. Note: The color change in the picture corresponds to the dispersion spectrum of visible light at the corresponding wavelength. (**e**) CIE 1931 diagram containing the colour coordinates of the O-LED, G-LED, B-LED and WLED devices.

The CDs were used in LEDs due to their unique optical properties, excellent colour stability and wide emission peaks (Figure S2). The three kinds of CDs were directly coated on UV-LED chips to obtain orange, green and blue LEDs (Figure 8a–c) [48,49,50,51]. Their optimal fluorescence centres are at 589, 535 and 457 nm, consistent with the solid fluorescence results of the original CDs. The CIE coordinates of the O-LED, G-LED and B-LED are (0.44, 0.48), (0.35, 0.52) and (0.18, 0.26), respectively (Figure 8e). A WLED was prepared by mixing the O-CDs, G-CDs and B-CDs on an LED-based chip in a ratio of 2:1:4 (Figure 8d). The PL emission spectrum of this WLED shows a panchromatic emission at 400–700 nm, and the corresponding fluorescence spectrum is consistent with the emission peaks of the three CDs. The overlap of different emissions centred at 582, 520 and 456 nm confirms that there is no self-absorption or significant energy transfer between CDs [52,53], showing CIE coordinates of (0.34, 0.31). We summarize the recent literature data on CD-based LED colour coordinates in Table S3 and compare it with the colour coordinates of LEDs in this study in Figure S3 [54,55], showing that the fabricated LEDs emit single orange, green, blue and white light, indicating the successful application of solid fluorescent CDs in LEDs.

## 3. Discussion

In summary, orange, green and blue solid fluorescent CDs were prepared in methanol, ethanol and water using citric acid, urea and phenylethylamine as raw materials. The PLQYs of the solid fluorescent CDs were 13.9%, 35.7% and 2.6%, respectively. We elucidated the solid-state fluorescence mechanism of the CDs. Phenylethylamine participates in the reaction to make the structure of the CD terminal connection have a spatial blocking effect. The carbon in the solid state, with a certain spacing between adjacent CDs, avoids direct contact between the luminescence centres, weakening the direct π–π interaction and the excessive Förster resonance energy transfer between them, thus achieving solid-state fluorescence. Different solvents influence the reaction degree of phenylethylamine, which makes the structure of phenylethyl connected to the surface of CDs different, resulting in a difference in the strength of the steric hindrance effect. Orange, green, blue and white LEDs with different CIE coordinates were produced based on the excellent optical properties of the CDs. This work provides a new method for the green and low-cost development of multicolour solid fluorescent CDs and shows great application potential in optoelectronic devices.

## 4. Materials and Methods

### 4.1. Materials

Citric acid (99.0%), urea (99.7%), phenylethylamine (99.0%), methanol (99.5%), ethanol (99.0%), supplied by Shanghai Tiantai Technology Co., Ltd. (Shanghai, China). All reagents are used directly upon receipt. Ultrapure water (Milli-Q water) prepared from Best-S15 system (Shanghai Zhion, Shanghai, China) with a resistivity of 18.2 MΩ cm was used in all experiments.

### 4.2. Methods

PL spectra were measured using a Shimadzu fluorescence spectrophotometer RF-6000 (Shimadzu, Tokyo, Japan). UV-vis spectra was collected with a Shimadzu UV-2600 spectrometer (Shimadzu, Tokyo, Japan). Transmission electron microscope (TEM) image of FEI Tecani G2 F20 (FEI, Hillsboro, OR, USA) measured at 200 kV accelerated voltage operation. Nanosecond fluorescence lifetime was measured by the time-correlated single photon counting (TCSPC) system (HORIBA Scientific iHR 320, Paris, France). Fourier transform infrared (FTIR) spectroscopy was achieved using a Thermo Scientific Nicolet iS5 spectrometer (Thermo Fisher Scientific, Waltham, MA, USA). X-ray photoelectron spectroscopy (XPS) was collected by K-alpha spectrometer (Thermo Fisher Scientific, Waltham, MA, USA). KONICA MINOLTA CS-150 colorimeter (Konica Minolta, Tokyo, Japan) was used to calibrate CIE colorimeter coordinates. Quantum yield (QY) was measured using an FLS1000 spectrometer (Techcomp, Livingston, Edinburgh, UK) equipped with a calibrated integrating sphere. 

### 4.3. Synthesis of CDs

Multicolour solid fluorescent CDs were prepared by using citric acid, urea and phenylethylamine as precursors and methanol, ethanol and water as solvents. In detail, 1.0 g citric acid and 1.0 g urea were dissolved in 10 mL water, and 1 mL phenylethylamine was added and ultrasonicated for 15 min. The mixture was then transferred to a 20 mL Teflon-lined stainless steel autoclave, heated to 180 °C, and maintained for 12 h. After cooling to room temperature, a blue fluorescent suspension was obtained. In addition, citric acid and urea were dissolved in methanol and ethanol, respectively, and 1 ml of phenylethylamine was added to prepare the other two CDs. The three CDs solutions were desiccated by vacuum steam and redissolved with dichloromethane. The crude products were purified by silica gel column chromatography using a mixture of dichloromethane and ethanol as the eluent. After solvent removal and further vacuum drying, three kinds of CDs were obtained. The yields of orange, green and blue CDs were 15.1 ± 1.9%, 18.2 ± 2.1% and 13.5 ± 1.6%, respectively. Under the same conditions, five repeated experiments were carried out on the preparation of three kinds of CDs, and fluorescence and UV characterization were carried out respectively, which showed that the prepared CDs had the same fluorescence characteristics, which proved that the preparation process of CDs has good repeatability.

### 4.4. Fabrication of LEDs

The emission peak of UV-LED chip is located at 365 nm and the operating voltage is 3.0 V. The tricolour CDs were dissolved in ethanol, respectively, and the resulting CDs’ solution (100 mg/mL) was dripped onto UV-LED chips, which were dried in an oven at 60 °C. Based on the solid fluorescence characteristics of CDs, blue, green and orange LEDs were realized. After the three CDs’ samples were mixed, the mixed CDs’ solution was dripped onto the UV-LED chip and dried in a 60 °C oven to obtain WLED.

## Figures and Tables

**Table 1:** Fluorescence characterization data of O-CDs, G-CDs and B-CDs.

Condition		O-CDs	G-CDs	B-CDs
Solution	λ_ex_ (nm)	549	543	440
	λ_em_ (nm)	460	460	370
	QY (%)	36.7	69.6	67.3
	τ_avg_ (ns)	7.73	7.87	4.57
Solid state	λ_ex_ (nm)	510	470	380
	λ_em_ (nm)	585	538	445
	QY (%)	13.9	35.7	2.6
	τ_avg_ (ns)	5.03	5.74	1.28

**Table 2:** XPS spectra data of O-CDs, G-CDs and B-CDs.

		O-CDs (%)	G-CDs (%)	B-CDs (%)
XPS survey	C1s	80.9	87.2	80.8
	N1s	10.9	5.0	1.8
	O1s	8.2	7.8	17.4
C1s	C=C/C–C	83.0	83.3	80.3
	C–N/C–O	12.3	10.0	11.4
	C=O	5.7	6.7	8.3
O1s	C=O	90.9	92.6	91.7
	C–O	9.1	7.4	8.3

## Data Availability

The data presented in this study are available on request from the corresponding author.

## Supplementary Materials

The following supporting information can be downloaded at: https://www.mdpi.com/article/10.3390/ijms231911071/s1.
