# Supplementary material for: Preparation of Multicolour Solid Fluorescent Carbon Dots for Light-Emitting Diodes Using Phenylethylamine as a Co-Carbonization Agent
Source: Int J Mol Sci. 2022 Sep 21;23(19):11071. doi: 10.3390/ijms231911071 (PMC9569546; doi:10.3390/ijms231911071)
Supplement: Supplementary file 1 [file ijms-23-11071-s001.zip › ijms-1910570-supplementary.pdf]

Supporting Information

Preparation of Multicolour Solid Fluorescent Carbon Dots for Light-Emitting Diodes Using Phenylethylamine As a Co-Carbonization Agent

Yulong An [1], Can Liu [1,2,*], Yan Li [1], Menglin Chen [1], Yunwu Zheng [1], Hao Tian [3], Rui Shi [2], Xiahong He [2] and Xu Lin[1,2,*]

[1] National Joint Engineering Research Center for Highly-Efficient Utilization Technology of Forestry Resources, Southwest Forestry University, Kunming 650224, China

[2] Key Laboratory for Forest Resources Conservation and Utilization in the Southwest Mountains of China, Ministry of Education, Southwest Forestry University, Kunming 650224, China

[3] Agro-products Processing Research Institute, Yunnan Academy of Agricultural Sciences, Kunming 650000, China

[*] Correspondence: liucan@swfu.edu.cn (C.L.); linxu@swfu.edu.cn (X.L.)

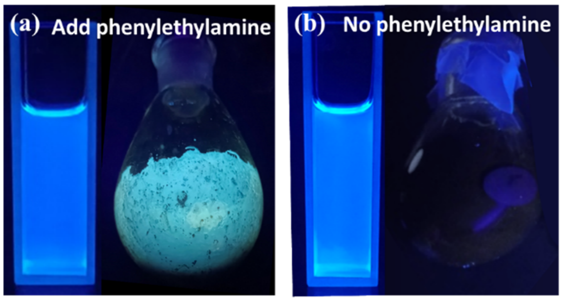

Figure S1. CDs prepared in reaction systems with phenylethylamine (**a**) and without phenylethylamine (**b**).

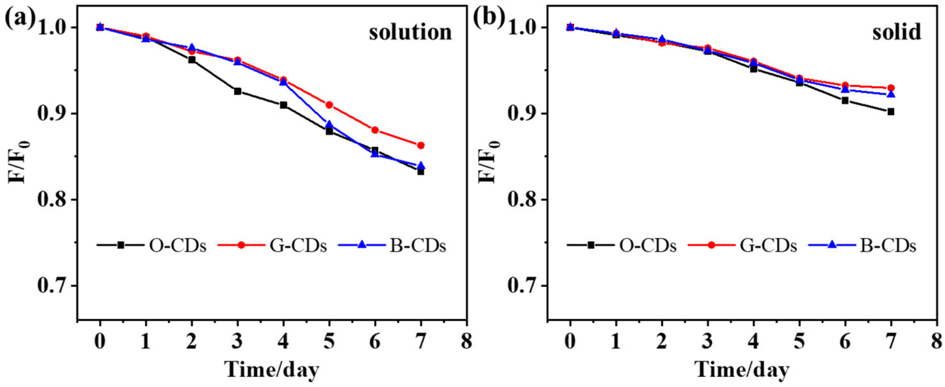

Figure S2. Decay curve of PL intensity of CDs in solution (**a**) and solid state (**b**) with increasing visible light time.

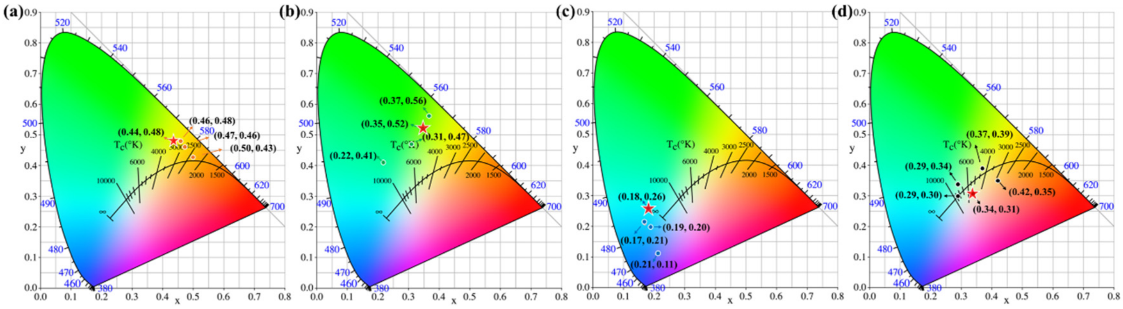

Figure S3. CIE 1931 diagram comparing the color coordinates of (**a**) orange, (**b**) green, (**c**) blue and (**d**) white LEDs reported in the literature with the LEDs in this study. Note: The red star represents the color-coordinate position of the LED prepared in this study.

Table S1. Comparison of process parameters and performance of O-CDs, G-CDs and B-CDs.

Sample	Synthetic Method		PL Color		Stability		QY	
	Reaction Precursor	Reaction Solution	Solution	Solid	Solution	Solid	Solution	Solid
O-CDs	Citric acid + urea + phenylethylamine	methanol	green	orange	83%	90%	36.7%	13.9%
G-CDs		ethanol	green	green	86%	93%	69.6%	35.7%
B-CDs		water	blue	blue	84%	92%	67.3%	2.6%

Table S2. The preparation method and fluorescence QY of solid fluorescent CDs have been reported.

Preparation Method	CDs PL Color	QY (%)	References
**Assemble $BaSO_4$ on the CDs surface**	green	27.0	[25]
**Scattered in PVP matrix**	red	41.3	[31]
**One step hydrothermal method**	white	2.0	[30]
**One step hydrothermal method**	green	26.0	[22]
**One step hydrothermal method**	white	35.0	[23]

Table S3. CIE coordinates of LEDs with different luminescence colors reported in the literature.

LED Color	CIE (X, Y)	References
**Blue**	0.17, 0.21	[14]
	0.21, 0.11	[16]
	0.19, 0.20	[54]
**Green**	0.22, 0.41	[15]
	0.31, 0.47	[54]
	0.37, 0.56	[55]
**Orange**	0.47, 0.46	[7]
	0.50, 0.43	[15]
	0.46, 0.48	[54]
**White**	0.37, 0.39	[14]
	0.29, 0.30	[16]
	0.29, 0.34	[23]
	0.34, 0.31	[24]
	0.42, 0.35	[47]
